# Development and structural determination of an anti-PrP^C^ aptamer that blocks pathological conformational conversion of prion protein

**DOI:** 10.1038/s41598-020-61966-4

**Published:** 2020-03-18

**Authors:** Tsukasa Mashima, Joon-Hwa Lee, Yuji O. Kamatari, Tomohiko Hayashi, Takashi Nagata, Fumiko Nishikawa, Satoshi Nishikawa, Masahiro Kinoshita, Kazuo Kuwata, Masato Katahira

**Affiliations:** 1 Institute of Advanced Energy, Kyoto University, Kyoto, 611-0011 Japan; 2 Graduate School of Energy Science, Kyoto University, Kyoto, 611-0011 Japan; 3 Department of Chemistry and Research Institute of Natural Science, Gyeongsang National University, Gyeongnam, 52828 South Korea; 4 Life Science Research Center, Gifu University, Gifu, 501-1193 Japan; 5 National Institute of Advanced Industrial Science and Technology, Ibaraki, 305-8566 Japan; 6 United Graduate School of Drug Discovery and Medical Information Sciences, Gifu University, Gifu, 501-1193 Japan

**Keywords:** Solution-state NMR, Nucleic acids, RNA, Prions

## Abstract

Prion diseases comprise a fatal neuropathy caused by the conversion of prion protein from a cellular (PrP^C^) to a pathological (PrP^Sc^) isoform. Previously, we obtained an RNA aptamer, r(GGAGGAGGAGGA) (R12), that folds into a unique G-quadruplex. The R12 homodimer binds to a PrP^C^ molecule, inhibiting PrP^C^-to-PrP^Sc^ conversion. Here, we developed a new RNA aptamer, r(GGAGGAGGAGGAGGAGGAGGAGGA) (R24), where two R12s are tandemly connected. The 50% inhibitory concentration for the formation of PrP^Sc^ (IC_50_) of R24 in scrapie-infected cell lines was ca. 100 nM, i.e., much lower than that of R12 by two orders. Except for some antibodies, R24 exhibited the lowest recorded IC_50_ and the highest anti-prion activity. We also developed a related aptamer, r(GGAGGAGGAGGA-A-GGAGGAGGAGGA) (R12-A-R12), IC_50_ being ca. 500 nM. The structure of a single R12-A-R12 molecule determined by NMR resembled that of the R12 homodimer. The quadruplex structure of either R24 or R12-A-R12 is unimolecular, and therefore the structure could be stably formed when they are administered to a prion-infected cell culture. This may be the reason they can exert high anti-prion activity.

## Introduction

Prion diseases are fatal neurodegenerative disorders including Creutzfeldt–Jakob disease in humans, bovine spongiform encephalopathy in cattle, scrapie in sheep and goats, and other diseases^1–6^. Many studies revealed that the conformational transition of prion protein (PrP) from a normal cellular form (PrP^C^) into an abnormal form (PrP^Sc^) is a key event in the pathogenesis of prion diseases. Although the mechanism of transition from PrP^C^ to PrP^Sc^ and the structure of PrP^Sc^ remain unknown, the structure of PrP^C^ was used for development of drugs against prion diseases. It was reported that some antibodies^7^, nanobody^8^, aptamers^9,10^ and chemical compounds^11–17^ against PrP^C^ successfully reduced the amount of PrP^Sc^ in cells persistently infected with the transmissible spongiform encephalopathy (TSE) agent.

**Figure 1:**
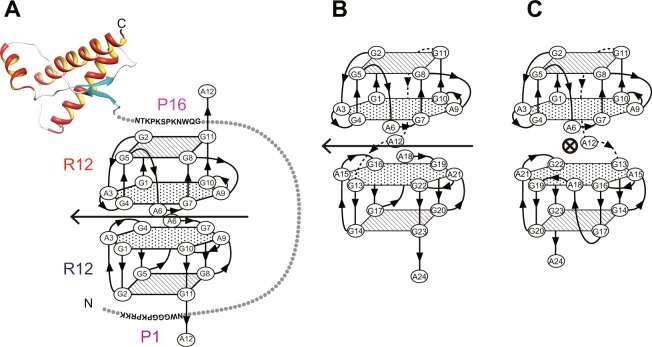
Schematic models of RNA aptamers. (**A**) R12 homodimer in complex with PrP^C^ ^20^. The structure of the C-terminal region of bPrP was drawn by using the coordinates of accession number 1DX0 of the Protein Data Bank^42^. (**B**,**C**) Two candidate architectures of R24. It is assumed that the A12 residue links two quadruplexes in a back to front (**B**) or a back to back manner. (**C**) The two-fold symmetrical axis that correlates two quadruplexes at the top and bottom is shown for each structure, respectively.

Previously we reported that an anti-PrP^C^ aptamer, r(G^1^G^2^A^3^G^4^G^5^A^6^G^7^G^8^A^9^G^10^G^11^A^12^) (R12), tightly binds to PrP^C^ and reduces the PrP^Sc^ level in mouse neuronal cells persistently infected with the human TSE agent^18–20^. It was revealed that R12 binds to two portions of bovine PrP (bPrP), P1 (residues 25–35 of bPrP) and P16 (residues 108–119 of bPrP), in the N-terminal intrinsically disordered region of PrP^19^. We also determined the structure of R12 in a complex with the binding peptide P16 (GQWNKPSKPKTN) to elucidate the mechanism by which R12 exhibits high affinity^20,21^. It was revealed that R12 folds into a unique quadruplex structure composed of a G:G:G:G tetrad plane and a G(:A):G:G(:A):G hexad plane, and that two R12 molecules bind in a tail-to-tail manner to form a homodimer. It was found that the R12 homodimer simultaneously binds to two portions of one PrP^C^ molecule (Fig. 1A), resulting in tight binding and stabilization of PrP^C^^20,21^. Here, we developed a new RNA aptamer, r(G^1^G^2^A^3^G^4^G^5^A^6^G^7^G^8^A^9^G^10^G^11^A^12^G^13^G^14^A^15^G^16^G^17^A^18^G^19^G^20^A^21^G^22^G^23^A^24^) (R24), containing two tandem R12 sequences. R24 was supposed to unimolecularly form a similar structure made of two R12 molecules. Therefore, under the conditions applied for the assay with prion-infected cells, it was expected that the structure of R24 is stably formed and that R24 exerts anti-prion activity to block the pathological conformational conversion of PrP. In fact, R24 showed much higher anti-prion activity than R12. Furthermore, another related aptamer, r(G^1^G^2^A^3^G^4^G^5^A^6^G^7^G^8^A^9^G^10^G^11^A^12^-A^13^-G^14^G^15^A^16^G^17^G^18^A^19^G^20^G^21^A^22^G^23^G^24^A^25^) (R12-A-R12), was also supposed to unimolecularly form a similar structure composed of two R12 molecules. It was revealed that R12-A-R12 also exhibited much higher anti-prion activity than R12, as expected. Then, we determined the structure of R12-A-R12 by NMR and deduced the mode of interaction of R12-A-R12 with PrP. These studies confirmed the origin of the high anti-prion activity of R12-A-R12 and R24, and provided further insight. The information on the sequences of potent aptamers and the structural bases of their anti-prion activity revealed in this study may be used to develop aptamer-based drugs against prion diseases.

## Results and Discussion

### Structure-based development of RNA aptamers having higher anti-prion ability to block the pathological conformational conversion of PrP than the R12 homodimer

We attempted to develop an RNA aptamer that exhibits higher anti-prion ability to block the pathological conformational conversion of PrP than R12 on the basis of the structure of R12. The unique quadruplex structure of a R12 monomer and its dimeric architecture are essential for tight binding to PrP^C^^20^. When R12 is dissolved in the cell culture for the assay, however, the R12 homodimer architecture may be destabilized, resulting in a decrease in the activity. Therefore, we tandemly connected two R12 monomers and obtained R24. R24 is expected to unimolecularly form a structure that resembles the structure formed by two R12 molecules. Two candidate structures were assumed for the overall architecture of R24 (Fig. 1B,C). In both cases, the positions of G1 - G11 of R24 are the same as those in the top R12 molecule of the R12 homodimer (Fig. 1A). In the Fig. 1B model, the positions of G13 - A24 of R24 are the same as those of G1 - A12 in the bottom R12 molecule of the R12 homodimer (Fig. 1A), as a result of the linkage of the two quadruplex units in a back side to front side manner via the A12 residue. On the other hand, in the Fig. 1C model, the A12 residue links the two quadruplexes in a back side to back side manner. The two-fold symmetrical axis that correlates two quadruplex units is shown in Fig. 1B,C, respectively. It should be noted that the latter architecture is the same as that found for d(GGA)_8_, whose structure was determined previously^22^. The real structure of R24 will be discussed later.

**Figure 2:**
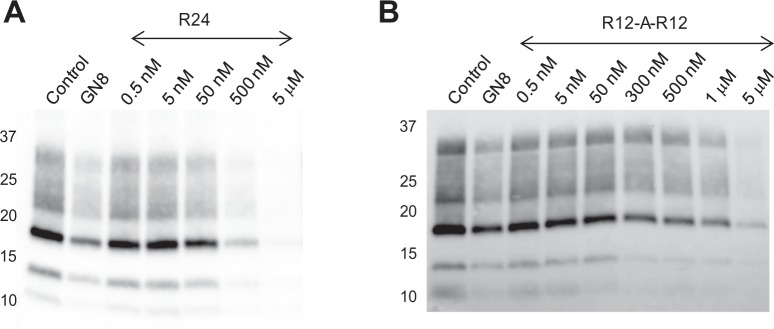
Anti-prion activity of R24 and R12-A-R12. Western blotting of PrP in GT + FK cells after the addition of various concentrations of either R24 (**A**) or R12-A-R12. (**B**) Lane 1 is a negative control, in which a buffer solution containing 100 mM KCl and 10 mM K-phosphate (pH 6.2) was added to the cell culture. Lane 2 is a positive control, in which 1.4 μM GN8, an anti-prion drug, was added. The experiments were carried out once for R24 and three times for R12-A-R12, respectively.

The anti-prion activity of R24 was examined using mouse neuronal cells (GT1-7) persistently infected with the human TSE agent (Fukuoka-1 strain), which was designated as GT + FK^12,15,17,23^. Either R24, an anti-prion drug, GN8 (positive control), or a solution containing 100 mM KCl and 10 mM K-phosphate (pH 6.2) (negative control) was added to the cell culture containing GT + FK12. R24 was found to significantly reduce the PrP^Sc^ level (Fig. 2A). 5 μM R24 added to the cell culture almost completely abolished the formation of PrP^Sc^ (Fig. 2A). Actually, the amount of PrP^Sc^ with 5 μM R24 was approximately 1% of that in the control. The 50% inhibitory concentration (IC_50_) for the formation of PrP^Sc^ of R24 was 100 nM (Supplementary Fig. S1A and Table 1). For R12, it was administered to the cell culture to a final concentration of 10 μM. Then, it turned out that the PrP^Sc^ level was reduced to 49 ± 17% of the control, that is to say, nearly 50% of the control. This suggests that the IC_50_ value of R12 is ca. 10 μM (Table 1). Thus, it is indicated that R24 possesses much higher, ca. 100-fold higher, anti-prion activity than R12. Furthermore, except for some antibodies^7^, R24 exhibited the lowest recorded IC_50_ determined by the prion-infected cell-based assay among all anti-prion materials involving nanobody^8^, RNA/DNA aptamers^9,10^, and chemical compounds^11–17^.

**Table 1:** 50% inhibitory concentrations (IC_50_) for the formation of PrP^Sc^ of anti-PrP^C^ aptamers.

Aptamer	IC_50_
R12^†^	(ca. 10 μM)^‡^
R24	97 nM
R12-A-R12	560 ± 148 nM

^†^With 20 units of RNase inhibitor.

^‡^See the main text for details.

The dissociation constant of the R12-bPrP complex was examined by filter binding assay^18,20^ and determined to be 18 nM^21^. The dissociation constant of the R24-bPrP complex was examined in the same way and determined to be 61 nM. The magnitude of the dissociation constant turns out to be the same order of 10^−8^ M for both R12 and R24.

**Figure 3:**
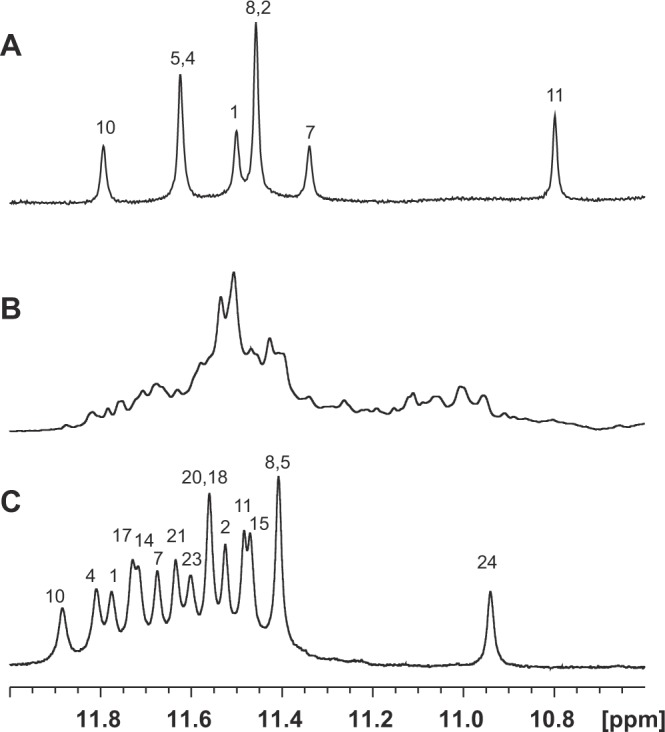
Imino proton spectra of anti-PrP^C^ aptamers: (**A**) r(GGAGGAGGAGGA) (R12), (**B**) r(GGAGGAGGAGGAGGAGGAGGAGGA) (R24) and (**C**) r(GGAGGAGGAGGA-A-GGAGGAGGAGGA) (R12-A-R12). Spectra were recorded in H_2_O solvent at 15 °C. The assignments are indicated by residue number.

We attempted structural analysis of R24 to confirm whether its structure resembles that of the R12 homodimer, as we expected. The imino proton signals of R24 were observed at 10.8–12 ppm (Fig. 3B), indicating the formation of a quadruplex. However, the quality of the NMR spectrum was not good enough for detailed structural analysis. To overcome this, we carried out screening to find an RNA that shows well-resolved imino proton signals among a series of RNAs with modified R24 sequences (Supplementary Table S1 and Fig. S2). As a result, an RNA sequence, R12-A-R12, was found to give a high-quality NMR spectrum (Fig. 3C). We then carried out an anti-prion assay using R12-A-R12, which demonstrated that R12-A-R12 can also reduce the PrP^Sc^ level significantly (Fig. 2B), with an IC_50_ value of ca. 500 nM (Supplementary Fig. S1B and Table 1). The IC_50_ of R12-A-R12 is of the same order of magnitude as that of R24, 10^−7^ M. Thus, it turned out that the potency of R12-A-R12 is also high. Therefore, we decided to carry out the structural analysis of R12-A-R12 as a representative RNA aptamer that exhibits much higher anti-prion activity than R12. It might also be a case that since the sequence of R12-A-R12 is almost the same as that of R24, the overall architecture of R12-A-R12 is expected to be similar, if not the same, as that of R24.

### The structure determination of R12-A-R12 by NMR

**Figure 4:**
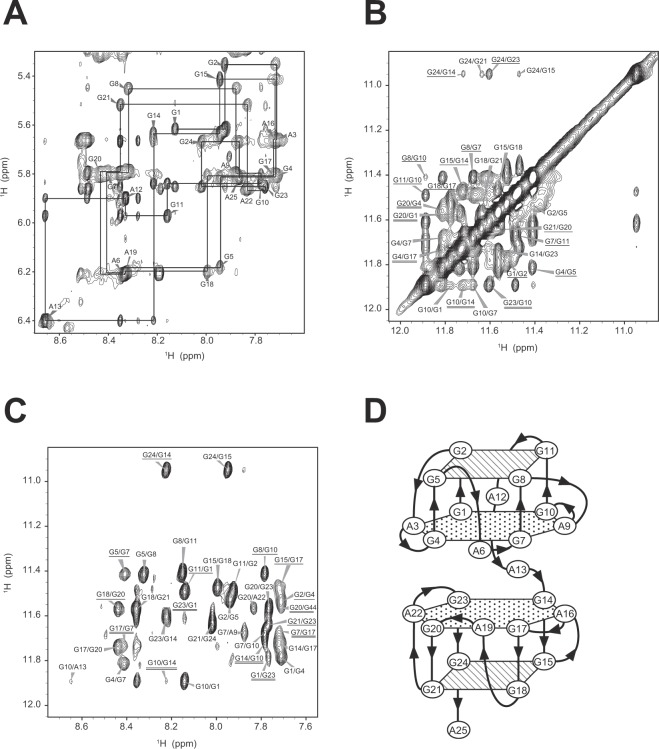
Assignments and identification of tetrad and hexad planes, together with the mode of hexad-hexad stacking, of R12-A-R12. (**A**) The fingerprint base proton/H1′ region of the NOESY spectrum in D_2_O solvent recorded with a mixing time of 400 ms at 15 °C. (**B**,**C**) Imino-imino (**B**) and imino-H8 (**C**) cross peaks of the NOESY spectrum in H_2_O solvent recorded with a mixing time of 400 ms at 5 °C. The cross peaks between the tetrad and hexad planes are underlined and those between two hexad planes are double-underlined. (**D**) The elucidated overall architecture of R12-A-R12.

To confirm that R12-A-R12 folds into a unimolecular structure that resembles the structure of the R12 homodimer, we performed NMR analysis of R12-A-R12. First of all, the line width for the structure of R12-A-R12 is almost the same as that for the homodimer structure of R12 (Fig. 3A,C). This indicates that the molecular weight of the structure of R12-A-R12 is almost the same as that of the homodimer structure of R12 comprising 24 residues in total. As R12-A-R12 comprises 25 residues, it was revealed that the structure of R12-A-R12 is a unimolecular one and that R12-A-R12 does not form a dimer. The resonance assignment was conducted in the same way as reported for R12^19,20^. The nonexchangeable protons were assigned by NOESY, TOCSY, and DQF-COSY experiments. Figure 4A shows sequential NOESY connectivities for fingerprint base proton/H1′. Imino protons were assigned by means of a JRHMBC experiment that gave NH (imino proton)-C5 and H8-C5 correlation peaks for each guanine base (G)^24^. The characteristic interresidue NOEs between GNH (NH of G)/GNH2 (amino protons of G) and GH8 (H8 of G), and those between GNH and GNH established the formation of four G:G:G:G tetrad planes for G2:G5:G8:G11, G15:G18:G21:G24, G1:G4:G7:G10 and G14:G17:G20:G23 (Fig. 4B,C). Since the latter two tetrad planes show NOEs between GNH/GNH2 and AH8 (H8 of adenine base (A)), and GNH2 and ANH2 (amino protons of A), the formation of G1(:A3):G4:G7(:A9):G10 and G14(:A16):G17:G20(:A22):G23 hexad planes was confirmed.

The interresidue AH2 (H2 of A)-AH1′ (H1′ of A) and AH2-AH8 NOEs between A3 and A22, and A9 and A16 suggest that the G1(:A3):G4:G7(:A9):G10 hexad plane stacks on the G14(:A16):G17:G20(:A22):G23 hexad plane, as shown in Fig. 4D. Additionally, this mode of stacking of two hexad planes is supported by identification of NOEs between G1 and G23, G4 and G20, G7 and G17, and G10 and G14.

**Figure 5:**
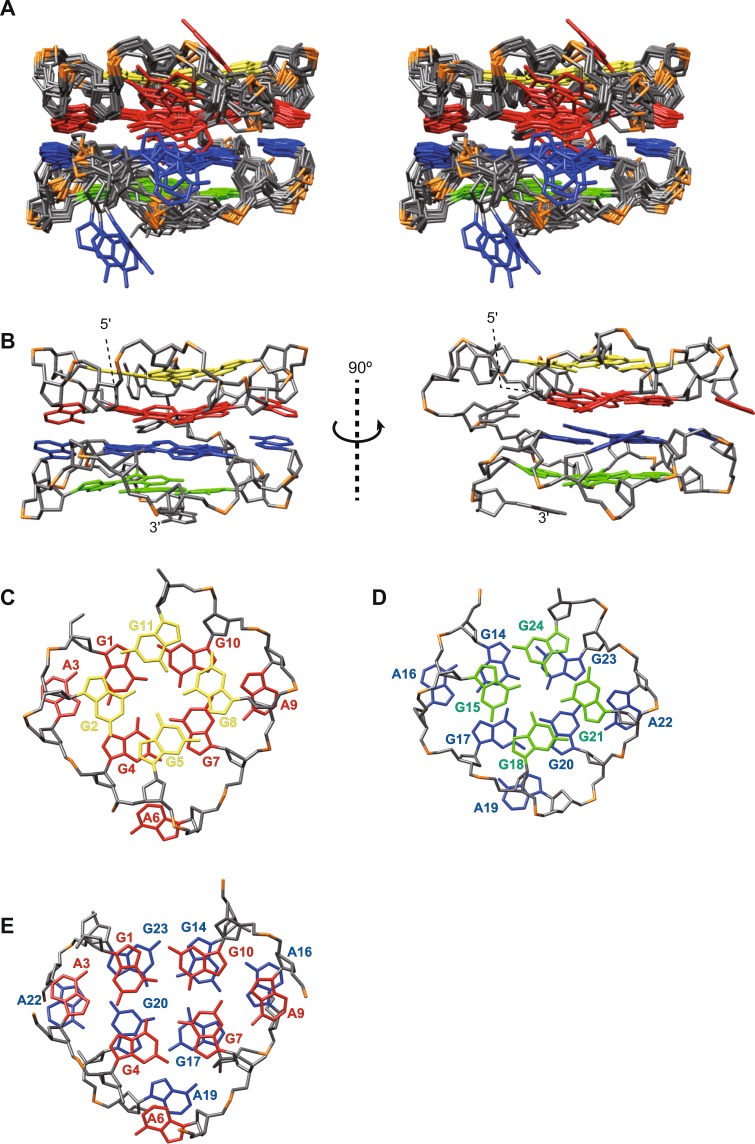
The structure of R12-A-R12. G2, G5, G8, and G11 (yellow), G1, A3, G4, A6, G7, A9, and G10 (red), G14, A16, G17, A19, G20, A22, and G23 (blue), G15, G18, G21, and G24 (green) are color coded. (**A**) A stereo view of the superimposed 10 refined structures. (**B**) The representative structure. (**C**) Stacking configuration of the G2:G5:G8:G11 tetrad and the G1(:A3):G4:G7(:A9):G10 hexad. (**D**) Stacking configuration of the G15:G18:G21:G24 tetrad and the G14(:A16):G17:G20(:A22):G23 hexad. (**E**) Stacking configuration of two hexads.

The structure of R12-A-R12 was calculated on the basis of NMR restraints obtained as described previously^19,22^ (Supplementary Table S2) by using the XPLOR-NIH program, as for R12^19,20^. Ten of the obtained lowest energy structures were further refined by NMR-based molecular dynamics (MD) simulations, which utilize NMR restraints with explicit water molecules by using the AMBER 16 program. The statistics for the ten refined structures are listed in Supplementary Table S2. The refined structure derived from the structure with the lowest XPLOR-NIH energy was selected as a representative structure. The structure of R12-A-R12 is shown in Fig. 5. R12-A-R12 comprises two tetrad and two hexad planes, whose arrangement is consistent with that shown in Fig. 4D. A6, A12, A13, A19 and A25 are not involved in either a tetrad or hexad plane. The two parallel quadruplexes within R12-A-R12 are packed in a tail-to-tail manner through stacking between two hexad planes (Figs. 4D and 5). Figure 5C–E show the stacking between planes. It should be noted that the bottom quadruplex of the R12 homodimer structure is rotated by 180° in the arrangement of the R12-A-R12 structure. Overall, it was revealed that one R12-A-R12 molecule forms a structure that resembles the structure of the R12 homodimer.

### The deduced modes of interaction of R12-A-R12 as well as R24 with PrP^C^

**Figure 6:**
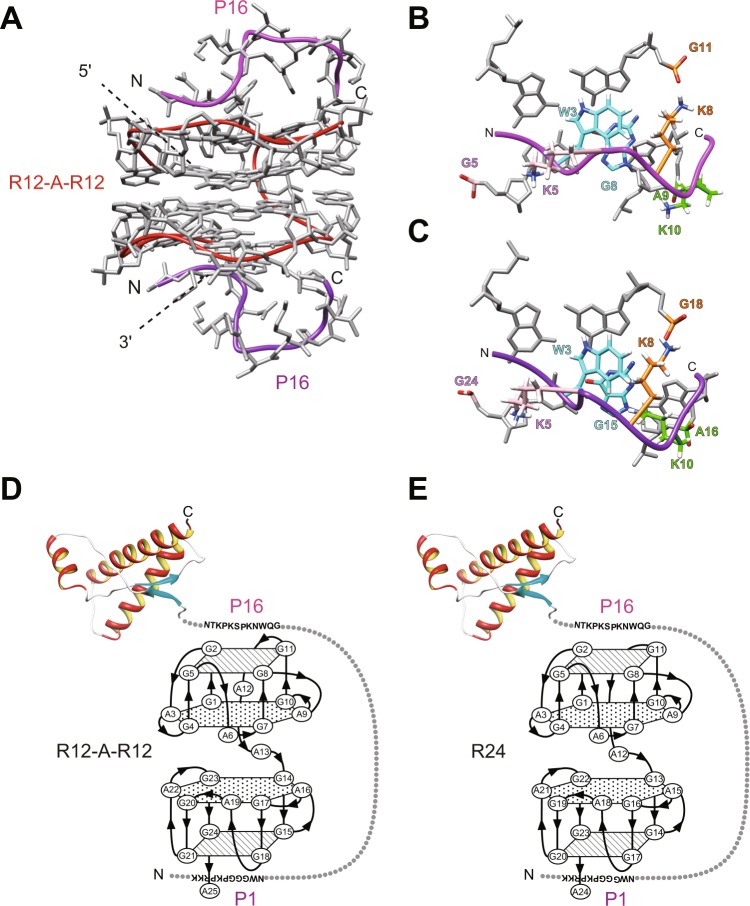
Models of either R12-A-R12 or R24 in a complex with PrP^C^. (**A**) The model structure of R12-A-R12 in a complex with two P16 (residues 108–119 of bPrP: GQWNKPSKPKTN) peptides. The structure was constructed by the replacement of the atomic coordinates of two R12 molecules in the R12-P16 complex (PDB ID: 2RU7) by those of one R12-A-R12 molecule. The red tube indicates the chain connecting the C5′ atoms of R12-A-R12. The magenta and purple tubes indicate the chain connecting the C_α_ atoms of each P16 monomer, respectively. (**B**) Close-up views of the interactions between R12-A-R12 and the upper P16 in the model. The G5 and K5, A9 and K10, G11 and K8, and G8 and W3 pairs are indicated by pink, green, orange and cyan, respectively. (**C**) Close-up views of the interactions between R12-A-R12 and the lower P16 in the model. The G24 and K5, A16 and K10, G18 and K8, and G15 and W3 pairs are indicated by pink, green, orange and cyan, respectively. (**D**,**E**) A schematic model of either R12-A-R12 (**D**) or R24 (**E**) in a complex with PrP^C^.

Previously, we determined the structure of the R12 homodimer in a complex with two molecules of the binding peptide of PrP, P16, by NMR^20,21^. Here, we constructed a model of a single R12-A-R12 molecule in a complex with two P16 peptides. The structure of R12-A-R12 determined in this study was superimposed on the previously determined homodimer structure of R12 in a complex with two P16 peptides and then the coordinates of the R12 homodimer were removed (Fig. 6A).

In the case of the R12 homodimer in a complex with two P16 peptides, we have recently pointed out that the effects of hydration are crucial in the binding and as a consequence the charge and shape complementarities within the binding interface play essential roles in stabilizing the complex^21^. Three Lys residues, K5, K8 and K10, of the P16 peptide came in contact with the phosphate groups of the G5, G11 and A9 residues of R12, respectively, reflecting the charge complementarity^20,21^. The stacking of a Trp residue, W3, of the P16 peptide and the G8 residue of R12 occurred as paradigmatic part of the shape complementarity^20,21^. These complementarities are basically conserved in the model of R12-A-R12 in a complex with two P16 peptides as well. First, the model is characterized by the contact of K5:G5, K8:G11 and K10:A9 and the stacking of W3:G8, though the K5:G5 distance is greater (Fig. 6B). Secondly, the model shows the contact of K5:G24, K8:G18 and K10:A16 and the stacking of W3:G15, though the K5:G24 distance is greater (Fig. 6C). These results indicate that the binding mode of a single R12-A-R12 molecule and two P16 peptides is physically the same as that of the R12 homodimer and two P16 peptides.

It was shown that the R12 homodimer binds to P16 and another binding peptide of PrP, P1, with almost the same affinity^20^. Therefore, for the R12 homodimer, we concluded that it tightly binds to PrP^C^ in the mode shown in Fig. 1A, resulting in the inhibition of the PrP^C^-to-PrP^Sc^ conversion^20^. It is reasonable to suppose that a single R12-A-R12 molecule tightly binds to PrP^C^ in the mode shown in Fig. 6D, resulting in the inhibition of the PrP^C^-to-PrP^Sc^ conversion. The same mode is also likely for R24 (Fig. 6E). It should be added that the structure of R24 in Fig. 6E corresponds to one of the two candidate architectures discussed above, Fig. 1C.

## Conclusion

In this study, we rationally designed new potent anti-PrP^C^ aptamers, R24 and R12-A-R12, on the basis of the structure of R12 and proved their high anti-prion abilities to block the pathological conformational conversion of PrP by means of a cell-based assay.

In order to elucidate the origin of high anti-prion activity, we determined the structure of R12-A-R12 by NMR. It was revealed clearly that a single R12-A-R12 molecule forms a unique quadruplex structure that resembles that of the R12 homodimer, as intended. Then, by use of the determined structure of R12-A-R12, a model of R12-A-R12 in a complex with two molecules of binding peptide P16 of PrP was constructed on the basis of the previously determined structure of the R12 homodimer in a complex with two P16 peptides. The constructed model indicated that R12-A-R12 can interact with and trap two P16 peptides, as the R12 homodimer does. Finally, it was deduced that the mode of the specific interactions of R12-A-R12 with PrP^C^ is similar to that of the R12 homodimer with PrP^C^.

Initially, we speculated that the unimolecular structure is stable even at the low RNA concentration, 10 μM, applied for the cell-based anti-prion assay, while the homodimer structure is not so stable at the low RNA concentration due to dissociation of the homodimer and that this could be a reason why RNA that can unimolecularly form the unique quadruplex structure exhibits higher anti-prion activity. However, the dissociation constant of R12-bPrP is 18 nM^21^. As the homodimer structure of R12 is essential for tight binding of PrP^20^, this result indicates that a certain amount of the homodimer structure still remains at the RNA concentration of 18 nM, which is much lower than 10 μM applied for the cell-based assay. Thus, the dissociation of the homodimer at the concentration applied for the cell-based assay cannot be a determinant to lower the anti-prion activity of R12.

When the R12 homodimer was administered to a prion-infected cell culture, the homodimer architecture of R12 may be destabilized and transiently destroyed by components in the culture. The transient destruction of the homodimer architecture may also cause the exposure of a fragile single-stranded R12 monomer to nucleases present in the cell culture and thereby degradation of the R12 molecule. The transient destruction and/or resultant degradation could cause the decrease in the anti-prion activity of R12. In contrast, the unimolecularly formed structure of R12-A-R12 is supposed to be more resistant to the transient destruction of the structure and/or resultant degradation in a cell culture, because two quadruplexes of R12-A-R12 are connected by covalent bonds. This is suggested to be the origin of the high anti-prion activity of R12-A-R12.

R24 exhibited even higher anti-prion activity. Except for some antibodies^7^, R24 exhibited the lowest recorded IC_50_ determined by the prion-infected cell-based assay among all anti-prion materials involving nanobody^8^, RNA/DNA aptamers^9,10^, and chemical compounds^11–17^. Thus, R24 is a promising anti-prion material. As the sequence of R24 is almost the same as that of R12-A-R12, it is likely that the structure and mode of the interaction with PrP^C^ of R24 are similar to those of R12-A-R12. The broadness of the imino proton spectrum of R24 hindered the structural determination of R24. This broadness may reflect the intermolecular aggregation of R24 with the high RNA concentration used for NMR analysis. In the case of a cell-based assay in which the RNA concentration is low, this possible aggregation is not likely to occur, which would be a reason why R24 exhibited high anti-prion activity like R12-A-R12. It was also noted that the dissociation constant of the R24-PrP complex is the same order of that of the R12-PrP complex. Resistance of the unimolecularly formed structure of R24 to the transient destruction and/or resultant degradation under cell culture may be why anti-prion activity of R24 is much higher than that of R12.

Although several anti-prion materials involving aptamers^9,10,25,26^, compounds^11–13,15–17^, and Fab^7^ have been reported, little is known about the structure and interaction at high resolution. The crystal structure was reported for a complex between a compound, promazine, and the C-terminal region of PrP^C^^27^. Crystal structures have also been reported for PrP^C^ in a complex with either an antibody^28,29^ or nanobody^8^. In all three cases, the C-terminal region of PrP was bound by each anti-prion material. The NMR structure has been reported only for the octapeptide portion of the N-terminal region of PrP^C^ in a complex with a polymer compound, pentosane polysulfate, the coordinates of pentosane polysulfate not being provided^14^. The structures and interactions of R12^20,21^ and R12-A-R12 are the only information reported for anti-prion aptamers at high resolution. This is also the only information reported for an anti-prion material that binds to the N-terminal region of PrP^C^ other than the octapeptide region. Therefore, the information obtained in this study is generally valuable for the development of potent anti-prion materials.

Alzheimer’s disease (AD) is one of the most common neurodegenerative disorders and the link between amyloid β (Aβ) and AD has been suggested^30,31^. It was reported that PrP^C^ that is anchored to the cell surface acts as a receptor of the Aβ oligomer^32^. The interaction of Aβ oligomers with PrP^C^ reportedly causes the neurotoxicity of Aβ oligomers such as inhibition of long-term potentiation^32^. Thus, it is thought that compounds that can disrupt the formation of the PrP^C^-Aβ oligomer complex may prevent AD. Recently, we reported that R12 can disrupt the PrP^C^-Aβ complex through tight binding of R12 to PrP^C^^33^. This result suggested the therapeutical potential of R12 for treating AD pathology. As R24 and R12-A-R12 tightly bind to PrP^C^ and exhibit higher anti-prion activity than R12 in the cell-based assay, they may also disrupt the PrP^C^-Aβ complex, even more efficiently than R12. Therefore, R24 and R12-A-R12 may have the therapeutical potential for treating not only prion but also AD pathology.

The development of drugs against prion diseases is waited right now. Nucleic acid-based drugs are widely recognized as next-generation ones. We demonstrated the way of rational developing of nucleic acids that exhibit much higher activity. Rational interpretation of the achieved much higher anti-prion activity is also provided on the basis of the structure determination. The substantial advance of the achievement of the low IC_50_ value of 100 nM opens the realistic possibility of the application of these RNAs for therapeutic use.

## Methods

### Sample preparation

R24 [r(GGAGGAGGAGGAGGAGGAGGAGGA)], R12-A-R12 [r(GGAGGAGGAGGA-A-GGAGGAGGAGGA)], and other RNAs with modified R24 sequences, that were synthesized, purified by high-performance liquid chromatography and desalted, were purchased from Japan Bio Services Co., Ltd. Oligonucleotides were dissolved in a solution comprising 10 mM KCl, 10 mM K-phosphate (pH 6.2), and 1.0 mM DSS, which was used as an internal chemical shift reference. The concentration of R12-A-R12 was ca. 1.0 mM for structural determination, and those of oligonucleotides were 50–100 μM for screening. Each sample was heated at 95 °C for 5 min, followed by quick cooling on ice.

### Evaluation of anti-prion activity

The anti-prion activity of either R24 or R12-A-R12 was examined using mouse neuronal cells (GT1–7) persistently infected with the human TSE agent (Fukuoka-1 strain), designated as GT + FK^12,15,17,23^, as reported previously^20^. The source is cells described in the report by Milhavet *et al*.^23^ Either R24 or R12-A-R12 was dissolved in a buffer solution containing 10 mM K-phosphate (pH 6.2) and 100 mM KCl. The samples were heated at 95 °C for 5 min, quickly cooled on ice and then stored at 4 °C before use. Approximately 1.5 × 10^5^ cells were plated in each well of a six-well plate, and drug treatment was started 15 h later. Either R24 or R12-A-R12 was administered to the cell culture to a final concentration of 0.5 nM, 5 nM, 50 nM, 500 nM or 5000 nM, respectively, and additionally to 300 nM and 1000 nM for R12-A-R12. The buffer and GN8 solutions were used as negative and positive controls, respectively. During incubation, it was confirmed by the observation and cell counting with microscope that the cells were alive and still attached to the bottom of the well after treatment with the RNA. After 72 h incubation, cells were lysed in 150 μl of 1×Triton X-100-deoxycholate lysis buffer containing 150 mM NaCl, 0.5% of Triton X-100, 0.5% of sodium deoxycholate and 50 mM Tris–HCl (pH 7.5), and then the supernatant was collected. Each sample concentration was adjusted to 2 mg of protein per milliliter. Samples were digested with 20 μg/ml of proteinase K for 30 min at 37 °C. Western blotting for PrP^Sc^ was performed as described previously^20^. For detection of PrP^Sc^, PrP M-20 antibody (Santa Cruz Biotechnology) was used as the primary antibody. The signals were visualized with Super-Signal (Pierce Biotechnology) and scanned using a LAS-1000 UV mini analyzer (Fuji Film). The experiments were carried out once for R24 and three times for R12-A-R12, respectively. The curves that allowed obtaining the IC_50_ values of R24 and R12-A-R12 are shown in Supplementary Fig. S1A,B, respectively. For R12, it was administered to the cell culture to a final concentration of 10 μM. After 72 h incubation, the PrP^Sc^ level was obtained in the same way. This experiment was carried out four times.

### Determination of the dissociation constant with filter binding assay

5′-end γ-^32^P-labeled R24 was mixed with varying concentrations of bPrP to give a total volume of 25 μl in a solution comprising 20 mM Tris-HCl (pH 7.5) and 10 mM KCl. After 20 min incubation, each mixture was passed through a nitrocellulose filter and washed with 500 μl of the solution. The amount of bound R24 was measured with BAS 2500 (Fuji Film), and binding activities were calculated as the percentage of input R24 in the bPrP-R24 complex retained on the filter. We determined the dissociation constant (K_d_) using GraphPad PRISM and non-linear regression curve fitting, and a one site-binding hyperbola equation (R24 binding (%) = B_max_ × [bPrP]/(K_d_ + [bPrP]), where B_max_ is the maximum bound at the saturating bPrP concentration)^18,20^.

### NMR spectroscopy

One-dimensional ^1^H NMR spectroscopy, nuclear Overhauser effect spectroscopy (NOESY), total correlation spectroscopy (TOCSY), double-quantum filtered correlation spectroscopy (DQF-COSY), ^1^H-^13^C heteronuclear single quantum coherence (HSQC), and heteronuclear multiple bond coherence with jump and return solvent suppression (JRHMBC) were performed at 5, 15 and 25 °C with Bruker AVANCE III HD 600 MHz and AVANCE III HD 950 MHz spectrometers equipped with a cryoprobe. NMR data were processed and analyzed with XWIN-NMR/TopSpin (Bruker), NMRPipe^34^ and Sparky^35^.

### Structure calculation

The distance restraints between protons were estimated from NOESY spectra with mixing times of 100 ms and 400 ms, as described previously^19,22^. Dihedral angle restraints for the δ, and endocyclic ν0, ν1, ν2, ν3 and ν4 torsion angles were derived from ^3^J_H1′-H2′_ and ^3^J_H3′-H4′_ couplings from DQF-COSY spectra as described previously^19,22^. Structural calculations were performed using these restraints including hydrogen-bonding restraints for G:G and G:A base pairs, and planarity restraints for the tetrad and hexad with a simulated annealing protocol supplied with XPLOR-NIH v. 2.26^36,37^, as described previously^19,20^. NMR restraints and structural statistics are shown in Supplementary Table S2. Ten structures that were selected from 100 calculations on the basis of the criterion of the smallest residual energy were further refined by MD simulation as described below.

### Structure refinement

The structure of R12-A-R12 obtained by XPLOR-NIH was further refined by all-atom MD simulations with explicit water molecules. Ten structures derived on structural calculation with XPLOR-NIH were employed as the initial structures for the MD simulation. An MD simulation with NMR restraints on the basis of the NPT ensemble at 298 K under periodic boundary conditions was performed using the AMBER16 program^38^. The force-field parameters adopted for RNA and water molecules were ff99OL3^39,40^ and TIP4P-FB^41^, respectively. The time step was set at 2.0 fs and a 50-ns simulation was carried out. Detailed protocols are described in Supplementary Methods.

### Building of a structural model of R12-A-R12 in a complex with P16 peptides

A refined structure that was derived through a MD simulation from the lowest XPLOR-NIH energy was superimposed on the structure of the R12 homodimer in a complex with two P16 peptides (PDB ID: 2RU7) using the Match Maker module of UCSF Chimera. At this time, the numbers of the residues constituting the tetrad and hexad of the bottom R12 molecule of the homodimer were changed to correspond to those of the respective residues at the same positions in the R12-A-R12 molecule. Then, the atomic coordinates of R12 homodimer were removed.

### Data deposition

The atomic coordinates for the ensemble of the ten refined structures of R12-A-R12 were deposited in the Protein Data Bank (PDB), and are available under accession code 6K84.

## Supplementary information

Supplementary information.
