# Supplementary material for: Development and structural determination of an anti-PrPC aptamer that blocks pathological conformational conversion of prion protein
Source: Sci Rep. 2020 Mar 18;10:4934. doi: 10.1038/s41598-020-61966-4 (PMC7080826; doi:10.1038/s41598-020-61966-4)
Supplement: Supplementary file 1 — Supplementary information. [file 41598_2020_61966_MOESM1_ESM.pdf]

Development and structural determination of an anti-PrP^C aptamer that blocks pathological conformational conversion of prion protein

Tsukasa Mashima[1,2], Joon-Hwa Lee[3], Yuji O. Kamatari[4], Tomohiko Hayashi[1], Takashi Nagata[1,2], Fumiko Nishikawa[5], Satoshi Nishikawa[5], Masahiro Kinoshita[1,2], Kazuo Kuwata[6], and Masato Katahira[1,2]

[1] Institute of Advanced Energy, Kyoto University, Kyoto 611-0011, Japan
[2] Graduate School of Energy Science, Kyoto University, Kyoto 611-0011, Japan
[3] Department of Chemistry and Research Institute of Natural Science, Gyeongsang National University, Gyeongnam 52828, South Korea
[4] Life Science Research Center, Gifu University, Gifu 501-1193, Japan
[5] National Institute of Advanced Industrial Science and Technology, Ibaraki 305-8566, Japan
[6] United Graduate School of Drug Discovery and Medical Information Sciences, Gifu University, Gifu 501-1193, Japan

Table S1. RNA sequences derived from R24 used for screening.

Name	Sequence	Length
No. 1	UGGAGGAGGAGGAGGAGGAGGAGGA	25
No. 2	AGGAGGAGGAGGAGGAGGAGGAGGA	25
No. 3	GGAGGAGGAGGAGGAGGAGGAGGU	24
No. 4	GGAGGAGGAGGUGGAGGAGGAGGA	24
No. 5	GGAGGAGGAGGAGGAGGAGGAGGa	24
No. 6	GGAGGAGGAGGaGGAGGAGGAGGA	24
No. 7	AAGGAGGAGGAGGAGGAGGAGGAGGA	26
No. 8	AGGAGGAGGAGGAGGAGGAGGAGGAA	26
No. 9	AAGGAGGAGGAGGAGGAGGAGGAGGAA	27
No. 10	AAGGAGGAGGAGGAGGAGGAGGAGGAAA	28
No. 11	AAAGGAGGAGGAGGAGGAGGAGGAGGAA	28
No. 12	AAAGGAGGAGGAGGAGGAGGAGGAGGAAA	29
No. 13	AAgGAgGAgGAgGAgGAgGAgGAgGAA	27
No. 14	AAGgAGgAGgAGgAGgAGgAGgAGgAA	27
No. 15	AAGgAGgAGgAGgAGGAGGAGGAGGAA	27
No. 16	AAGGAGGAGGAGGAGgAGgAGgAGgAA	27
No. 17	AAGgAGGAGGAGgAGgAGGAGGAGgAA	27
No. 18	AAGGAGgAGgAGGAGGAGgAGgAGGAA	27
No. 19	AAGGAGGAGGAGGaGGAGGAGGAGGAA	27
No. 20	AAGGAGGAGGAGGagGAGGAGGAGGAA	27
No. 21	AAGGAGGAGGAGgaGGAGGAGGAGGAA	27
No. 22	AAGGAGGAGGAGgagGAGGAGGAGGAA	27
No. 23	AAGGAGGAGGAGGAAGGAGGAGGAGGAA	28
R24	GGAGGAGGAGGAGGAGGAGGAGGA	24
R12-A-R12	GGAGGAGGAGGAAGGAGGAGGAGGA	25

DNA nucleotides are indicated by lower-case letters.

Table S2. NMR restraints and statistics of ten refined structures of R12-A-R12.

**NMR distance and dihedral angle restraints**	
Distance restraints	903
Intra-residue	442
Inter-residue	381
Sequential (|i-j| = 1)	175
Non-sequential (|i-j| > 1)	206
Hydrogen bonds	80
Dihedral angle restraints	114
δ	19
ν_0-ν_4	95
**Structure statistics**	
Violations (mean and s.d.)	
Distance restraints (Å)	0.044 ± 0.000
Dihedral angle restraints (°)	0.299 ± 0.107
Max. distance restraints violation (Å)	0.466
Max. dihedral angle restraints violation (°)	4.707
Deviations from idealized geometry	
Bond lengths (Å)	0.011 ± 0.000
Bond angles (°)	2.286 ± 0.051
Impropers (°)	4.256 ± 3.089
Average pairwise r.m.s.d. (Å)	
All heavy atoms	1.390 ± 0.341
Heavy atoms of tetrad and hexad planes	0.761 ± 0.099

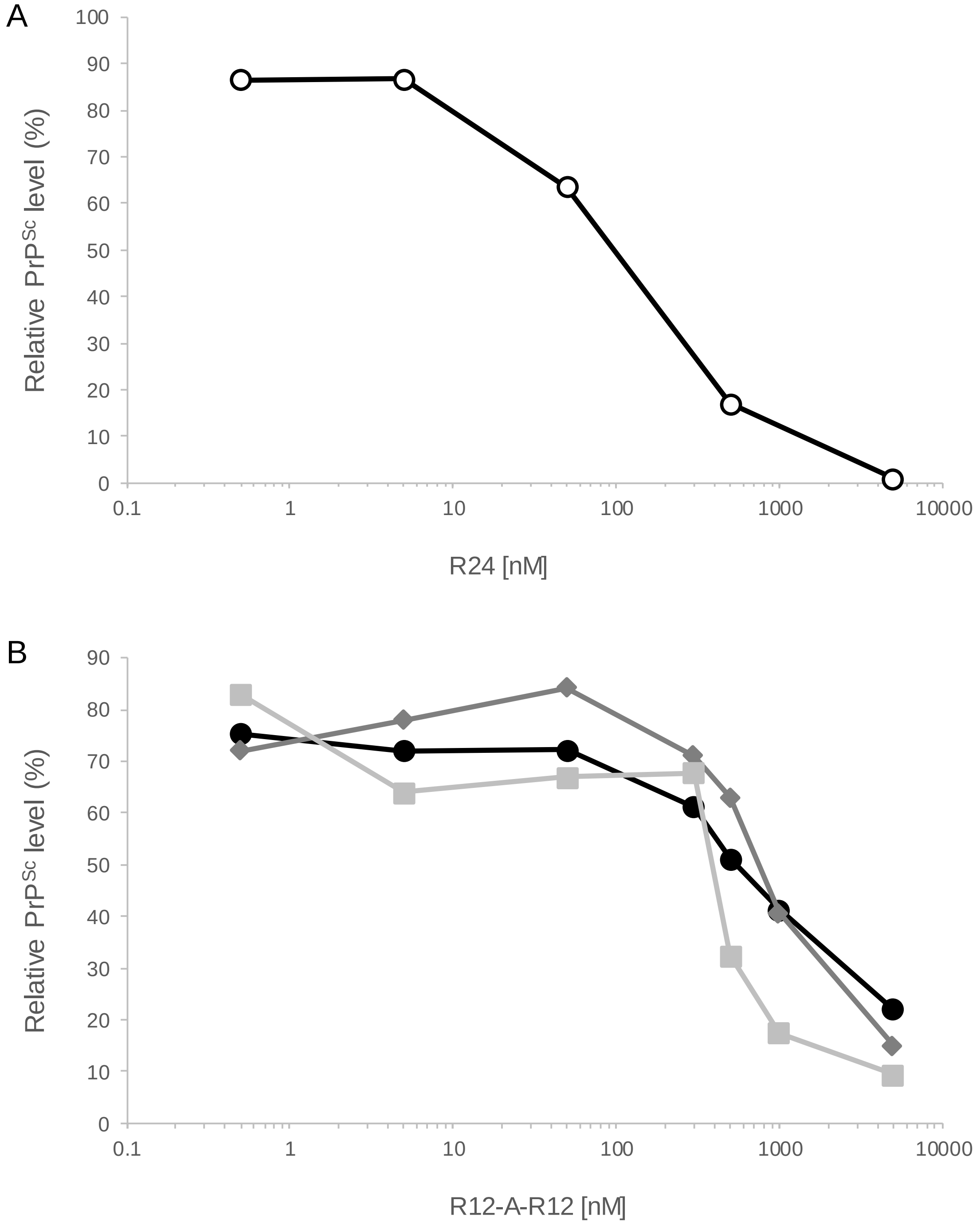

Fig. S1. Anti-prion activity of R24 and R12-A-R12. The relative PrP^{Sc} level is plotted against the applied concentration of either R24 (A) or R12-A-R12 (B). Experiments were performed once for R24 and three times for R12-A-R12.

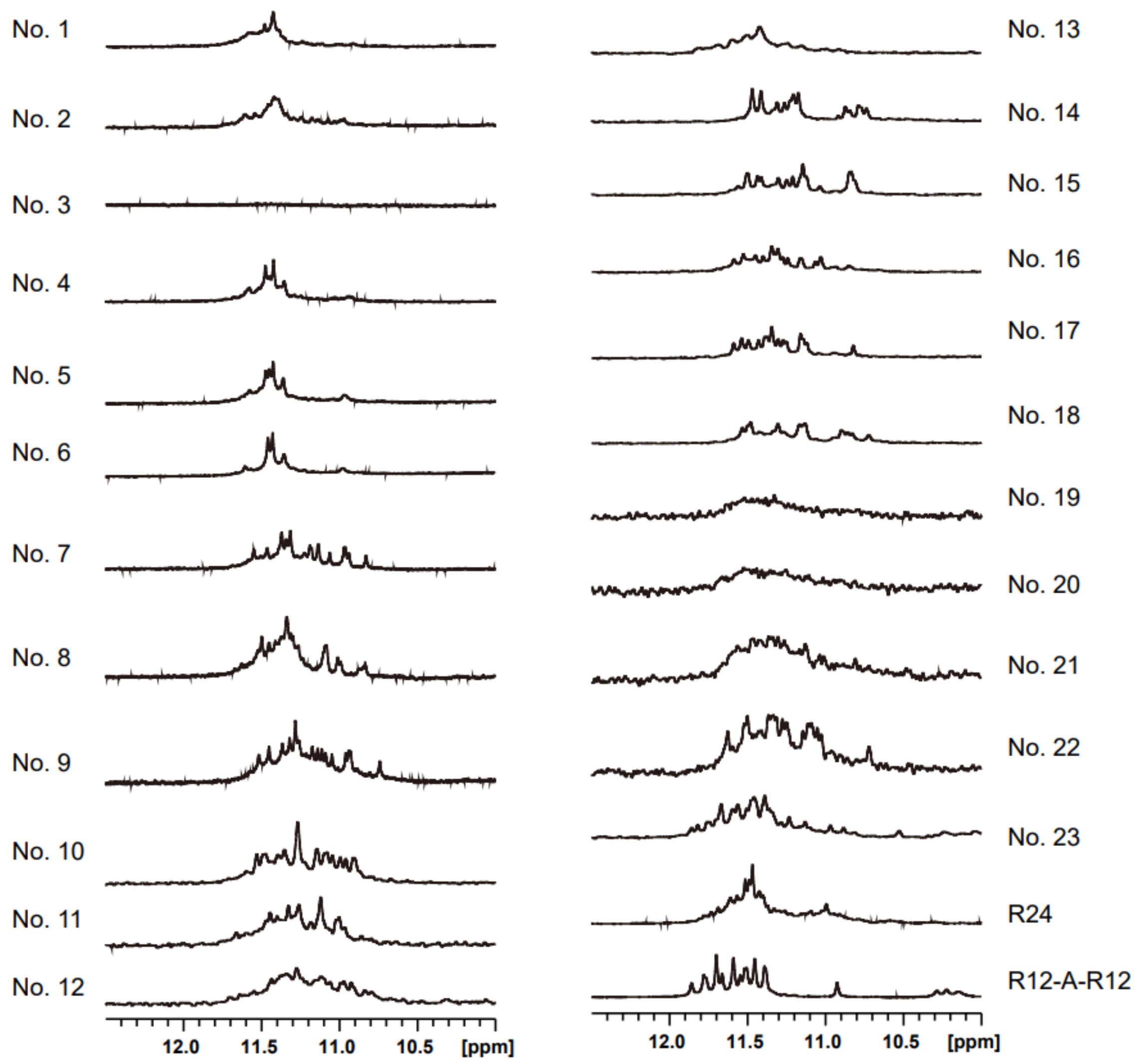

Fig. S2. Imino proton spectra of RNAs. Spectra were recorded in H_2O solvent at 25°C.

Supplementary methods

Structure refinement for R12-A-R12 using molecular dynamics simulations with NMR restraints

1 Simulation procedure

The structure of an R12-A-R12 monomer was refined using all-atom molecular dynamics (MD) simulations with explicit water. Ten of the NMR structures (hereafter, referred to as models 1 through 10) determined with the XPLOR-NIH program were employed as the initial structures. Using the AMBER16 program and starting from an initial structure, we performed an MD simulation with NMR restraints on the basis of the NPT ensemble at 298 K under a periodic boundary condition. Thus, a total of ten simulations were individually carried out. The solute (i.e., R12-A-R12) was immersed in water accommodated in the simulation box. The force-field parameters adopted for RNA and water molecules were ff99OL3 and TIP4P-FB, respectively. The minimum distance between the solute surface and box edge was initially set at ~15 Å, which was about 5.4 times larger (i.e., sufficiently larger) than the molecular diameter of water, 2.8 Å. The number of water molecules deviated significantly from model to model, ranging from 6947 to 8026, due to conformational diversity in the initial solute structure. In our NMR experiments, the aqueous solution contained KCl at 0.01 M/L. This very low concentration corresponded to the presence of two K^+ ions and two Cl^- ions in about 10,000 water molecules. In the MD simulation for R12-A-R12, whose total charge is −24e (e is the elementary charge), we added 24 Na^+ ions to the box to neutralize the system. The time step was set at 2.0 fs, and the lengths of bonds with H-atoms were constrained with the SHAKE algorithm. Electrostatic interaction was calculated using the particle-mesh Ewald (PME) method. The real-space cut-off, spline order, and Ewald tolerance for the PME method chosen were 10 Å, 4, and 10^{-5}, respectively. The cut-off for the Lennard-Jones (LJ) potential was set at 10 Å. The Langevin thermostat and Monte Carlo barostat were employed for regulating temperature and pressure, respectively.

We emphasize that all of the steps described below were performed with NMR restraints derived from NOE, J-coupling, and hydrogen bonding data. The planarity restraint for the tetrad or hexad structure in an R12-A-R12 monomer was also taken into account. Hereafter, these four types of restraints are put together and referred to simply as “NMR restraints”. Refer to Section 1.2 for the details of the methods for imposing the NMR restraints.

The temperature and pressure equilibration for the system was performed to obtain the NPT ensemble at 298 K and 1 atm. First, to remove the overlaps of the solute and water molecules, the initial positions of water molecules were slightly modified using the steepest descent and conjugated gradient methods, in which harmonic positional restraints were additionally applied to the solute atoms. The force constant for the restraints was set at 10 kcal/(mol·Å^2). By this modification, the potential energy of the

system became sufficiently low. We then applied positional restraints with 10 kcal/(mol·Å^2) to the solute heavy atoms, and water molecules were allowed to move during the subsequent temperature and pressure equilibration. We generated the velocities in accordance with a Maxwell distribution at 298 K and performed the temperature equilibration for 100 ps under the NVT condition. The pressure equilibration was then carried out at 298 K and 1 atm for 100 ps under the NPT condition. The solute structure thus obtained is referred to as the "pre-equilibrated structure". The positional restraints on the solute were then removed, NMR restraints being retained. Starting from the pre-equilibrated structure for each model, we performed a 50-ns simulation with the NPT ensemble at 298 K. Figure S3 shows the root mean square deviation (RMSD) from the pre-equilibrated structure in terms of all the solute atoms observed during the simulation. The fluctuation range was maintained within 0.5 Å for all the 10 models in 20 ns. After the 50-ns simulation, the final snapshot solute structure was taken and slightly modified to remove the distortion in the planarity of each single guanine or adenine nucleobase. This type of structure distortion arose from the NMR restraints imposed. We performed the steepest descent and conjugated gradient methods in the generalized Born implicit solvent by applying harmonic positional restraints with 100 kcal/(mol·Å^2) to the solute heavy atoms except those in the guanine or adenine nucleobase. The NMR restraints were retained during the slight modification. We note that the modified structure was essentially the same as the final snapshot structure obtained in the simulation. Thus, we constructed a total of ten refined structures.

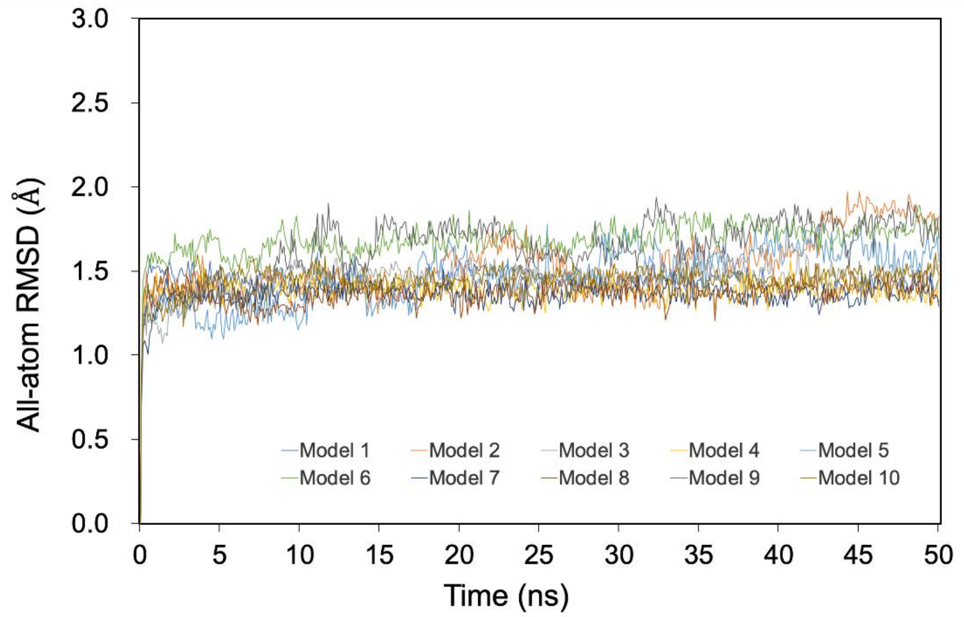

Fig. S3. Variation of the root mean square deviation (RMSD) from the pre-equilibrated structure of a R12-A-R12 monomer in terms of all the constituent atoms in the molecular dynamics simulation for each model.

2 Planarity restraints

Throughout the refinement, we applied a force of square bottom well possessing parabolic sides with a force constant of 20.0 kcal/(mol·Å^2) so that the distance restrains could be kept. The sugar pucker (torsional angle) restraints were also kept by applying the similar force with a force constant of 500.0 kcal/(mol·rad^2).

An R12-A-R12 monomer is characterized by two stacked hexad planes sandwiched by two tetrad planes. In a tetrad or hexad plane, four or six nucleobases are arranged on the same plane. To assure this characteristic, we imposed a planarity restraint on the structure refinement using the MD simulation as follows. Additional improper torsion angles were defined and implemented as illustrated in Figure S4. Throughout the refinement, we applied force to keep these improper torsion angles. The force constant in the application was set at 100 kcal/(mol rad^2). Though the solute structure fluctuated to a large extent in the MD simulation due to thermal fluctuation of the solute itself and thermal motion of water molecules, the nucleobases in each tetrad or hexad plane were arranged almost on the same plane after the refinement. The distance and sugar pucker (torsional) restraints were found to be kept within discrepancies of 0.5 Å and 5 degrees, respectively.

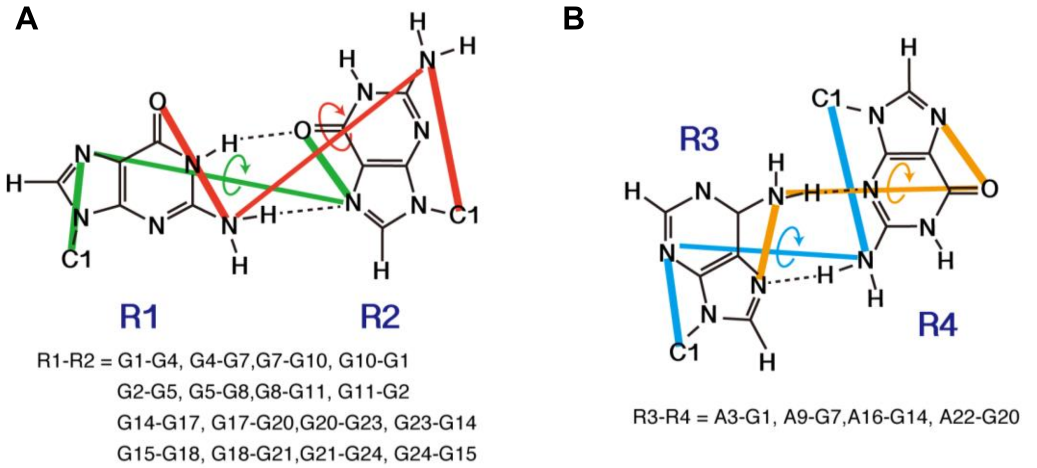

Fig. S4. Improper torsion angles (arrow-circles) for planarity restraint: (A) for Guanine-Guanine (G-G) pairs, and (B) for Adenine-Guanine (A-G) pairs. This figure also indicates the combinations of nucleobases to which the restraints were applied. The three green lines, for example, represent the following: The restraints are imposed so that the four atoms, C1, N, N, and O, are placed on the same plane.
